# Altitudinal changes induce responses in *Coptis chinensis* Franch. rhizomes: endophytic communities, metabolite types, and alkaloid contents

**DOI:** 10.3389/fpls.2026.1777206

**Published:** 2026-02-23

**Authors:** Wenli Zhang, Yanan Tang, Ran Luo, Jiang He, Jie Yan, Fei Long, Longyun Li

**Affiliations:** 1 Chinese Medicine Germplasm Resources Innovation and Effective Uses Key Laboratory of Sichuan Province, Key Laboratory of Standardization of Chinese Medicine, Ministry of Education, School of Pharmacy, Chengdu University of Traditional Chinese Medicine, Chengdu, China; 2 Chongqing Academy of Chinese Materia Medica, Chongqing, China; 3 College of Horticulture and Landscape Architecture, Southwest University, Chongqing, China

**Keywords:** altitude, community structure diversity, *Coptis chinensis* Franch., differentially expressed metabolites, LC-MS, metagenome

## Abstract

*Coptis chinensis* Franch. is a perennial medicinal plant with huge economic and social benefits, but how altitude affects the accumulation of bioactive compounds through microbial ecosystems remains unexplored. This study examined how microbial communities at different altitudes influence the bioactive components of *Coptis chinensis*, to help identify beneficial microorganisms for application to its rhizomes. Samples of *Coptis chinensis* were cultivated at four different altitudes in Shizhu, Chongqing. To characterize the phytochemical profile of *Coptis chinensis*, nine specific alkaloids were quantified by High Performance Liquid Chromatography (HPLC) and Ultraviolet-Visible Spectrophotometry (UV-Vis), with Liquid Chromatography-Mass Spectrometry (LC-MS) subsequently employed to characterize differential metabolite accumulation at each altitude. Microbial community structure in the rhizomes was analyzed by metagenomic sequencing. Results indicated that the contents of groenlandicine, coptisine, berberine, and total alkaloids increased with altitude, with the total alkaloid content rising from 15.97% at 907 m to 17.82% at 1698 m (*P* < 0.01). Analysis revealed 912 differential metabolites, with distinct accumulation patterns at different altitudes. Microbial diversity in the rhizomes also varied by altitude, with significant shifts in Mucoromycota, Pseudomonadota, *Rhizophagus*, and *Mesorhizobium* populations. Moreover, the relative abundance of these microorganisms was intricately linked to alkaloid content. High altitude significantly enhances alkaloid accumulation in *C. chinensis*, and this effect is primarily mediated by the enrichment of beneficial endophytes, which promote the biosynthesis of target alkaloids via optimizing nitrogen utilization and inducing the expression of key enzymes.

## 1 Introduction

*Coptis chinensis* Franch. (*C. chinensis*) is a perennial medicinal plant belonging to the Ranunculaceae family. Its rhizome is a well-known Chinese materia medica, commonly referred to as “weilian” or “jizhaolian, “ and has been extensively applied in traditional medicine with huge economic and social benefits. It is clinically applied to treat symptoms such as abdominal fullness, vomiting, acid regurgitation, diarrhea, boils, abscesses, and excessive heart fire (Li et al., 2023c). Some research has identified multiple bioactive components in *C. chinensis*, including alkaloids, lignans, flavonoids, and phenylpropanoids. These compounds are associated with blood glucose-regulating, antibacterial, anti-inflammatory, antiepileptic, and antitumor actions (Cui et al., 2018; Huang et al., 2022; Li et al., 2023b; Liu et al., 2023a; Wang et al., 2024; Yang et al., 2024b). *Shennong’s Classic of Materia Medica* (Volume Three) records that *C. chinensis* prefers shaded environments with low temperatures and high air humidity, growing in forested areas or shaded valleys at elevations between 500 and 2000 meters, with the optimal range being 1000 to 1800 meters (Wu, 1955). Currently, the main cultivation regions of *C. chinensis* are distributed within the latitude range of 28°N to 30°N, at elevations of 1200–1800 meters, with core production areas in Shizhu (Chongqing) and Lichuan (Hubei).

Plant growth and development are jointly regulated by multiple factors, including genotype, altitude, humidity, and the distribution of plant-associated microorganisms (Dai et al., 2025; Gao et al., 2025). Among these, altitude, as a synthetic environmental element, can regulate root metabolism, thereby influencing plant yield and quality (Zhou et al., 2021; Li et al., 2023a). For instance, the flavonoid content in *Ginkgo biloba* leaves increases with increasing altitude, and there are significant differences in endophytes in leaves at different altitudes (Fu et al., 2022); the output and pharmaceutical quality of wild *Artemisia argyi* populations are higher when grown below 500 m (Yang et al., 2024a); and altitude affects soil pH, trace element accumulation, and phenolic compound metabolism in *Asarum* (Pan et al., 2023).

Endophytes colonize inside rhizome tissues for a long time, forming a close symbiotic relationship with the host, and can directly participate in core physiological processes such as plant metabolic synthesis and stress resistance signal transduction (Terletskaya et al., 2024; Zhao et al., 2024). In addition, endophytes can prevent and control plant diseases and promote plant growth by inducing host resistance, competing with and inhibiting pathogens, etc (Collinge et al., 2022). Altitude can regulate the species composition of endophytes, mediate the positive correlative interactions between specific functional endophytes and metabolites, and thereby promote the adaptation of medicinal plants to high-altitude environments (Zhao et al., 2023). Nevertheless, how the *C. chinensis* endophytes vary with altitude has not been reported.

Although prior research has established that altitude affects the growth environment and quality of *C. chinensis*, such investigations have primarily focused on yield-related traits and individual chemical components (Zhao and Du, 2002; Zhou, 2013; Liu et al., 2014; Zhang et al., 2019). It is currently unclear whether the growth, bioactive component contents, and secondary metabolites of *C. chinensis* are affected by altitude. Therefore, this study investigated the diversity of secondary metabolites, variations in bioactive component contents across different altitudes, and the response of rhizome endophytes to altitude, aiming to clarify the influence of altitude on the medicinal parts of *C. chinensis*. Furthermore, this study established associations between *C. chinensis* metabolites and rhizome endophytes to reveal the tripartite relationship among the metabolites of *C. chinensis*, the diversity and abundance of endophytes, and the altitude factor.

## 2 Materials and methods

### 2.1 Sample collection and chemicals

Samples of *C. chinensis* were cultivated in Shizhu, Chongqing (29°39′-30°33′ N, 107°59′-108°34′ E), which features extensive mountainous terrain, fertile soil, lush vegetation, abundant rainfall, a cool climate, low sunshine exposure, and a short frost-free period, making it one of the major producing areas of *C. chinensis* (Zhao et al., 2025). Within the study area, under the conditions of the same mountain range and uniform management practices, four sampling sites were established along different altitudes, with the soil at all sampling sites being yellow-brown loam. All plants shared the same genetic background, the same cultivation batch, and the same growth period (5-year growth period), and three replicate samples were set at each site (presented in **Table 1**). All plant samples were identified as *C. chinensis* Franch. (Ranunculaceae) by Researcher LI Longyun from Chongqing Academy of Chinese Materia Medica.

**Table 1:** Sampling record of *C. Chinensis*.

Serial no.	Location	Planting mode	Altitude	Sampling time
HSA	Henong Village, Jinzhu Township, Shizhu County, Chongqing Municipality	high shed	907 m	November 2024
HSB	Henong Village, Jinzhu Township, Shizhu County, Chongqing Municipality	high shed	1271m	November 2024
HSC	Panlong Village, Shazi Town, Shizhu County, Chongqing Municipality	high shed	1556m	November 2024
HSD	Panlong Village, Shazi Town, Shizhu County, Chongqing Municipality	high shed	1698m	November 2024

Plant samples were divided into two portions. One portion comprised rhizomes: the surface of the rhizome tissues was cleaned with Wahaha purified water, followed by disinfection with 75% ethanol for 5 minutes, and then stored in liquid nitrogen. The other portion was dried at 60°C upon returning to the laboratory. The resulting material was powdered and screened through a 150-mesh sieve. The moisture content of the processed samples was determined to be < 10%, and they were stored for subsequent determination of chemical components in *C. chinensis*. Rhizosphere soil was collected from each sampling site. After moisture determination, the soil was air-dried, passed through a 2 mm sieve, stored at room temperature, and subsequently used for the determination of soil physical and chemical properties.

Instruments, reagents, and reference standards information were shown in **Tables 2**–**4**.

**Table 2:** Instrument information.

English name and model	Source company (Origin)
UV-1800 ultraviolet-visible spectrophotometer	Shimadzu Corporation (Japan)
Agilent 1260 high-performance liquid chromatograph	Agilent Technologies, Inc. (USA)
BioTek Flx800 microplate reader	BioTek Instruments, Inc.
KQ-250DB numerical control ultrasonic cleaner	Kunshan City Ultrasonic Instrument Co., Ltd.
XY-100MW-A halogen moisture meter	Changzhou City Xingyun Electronic Equipment Co., Ltd.

**Table 3:** Reagent information.

English name (Grade)	Source company (Origin)
Methanol (HPLC grade)	TEDIA Company (USA)
Acetonitrile (HPLC grade)	TEDIA Company (USA)
Hydrochloric acid (AR grade)	Chongqing Chuandong Chemical Co., Ltd.
Methanol (AR grade)	Chongqing Chuandong Chemical Co., Ltd.
Potassium chloride (AR grade)	Sinopharm Chemical Reagent Co., Ltd.
Ammonia water	Shandong Keyuan Biochemical Co., Ltd.
Triethylamine	Shandong Keyuan Biochemical Co., Ltd.
Ammonium bicarbonate	Shandong Keyuan Biochemical Co., Ltd.
Acetonitrile (LC-MS grade)	Fisher Scientific (Loughborough, UK)
Formic acid	TCI (Shanghai, China)
Ammonium formate	Sigma-Aldrich (Shanghai, China)
Wahaha purified water	Wahaha Brand

**Table 4:** Reference standards information.

Reference standards	Batch number	Purity	Source company
Magnoflorine	CYR-M0011210728	≥ 98%	Sichuan Cuiyi Run Biotechnology Co., Ltd.
Groenlandicine	CYR-G0046220901	≥ 98%	Sichuan Cuiyi Run Biotechnology Co., Ltd.
Jatrorrhizine	CYR-Y0011231201	≥ 98%	Sichuan Cuiyi Run Biotechnology Co., Ltd.
Columbamine	CYR-F0034231201	≥ 98%	Sichuan Cuiyi Run Biotechnology Co., Ltd.
Epiberberine	CYR-B0043220609	≥ 98%	Sichuan Cuiyi Run Biotechnology Co., Ltd.
Coptisine	CYR-H0057220202	≥ 98%	Sichuan Cuiyi Run Biotechnology Co., Ltd.
Palmatine Hydrochloride	CYR-H0076231201	≥ 98%	Sichuan Cuiyi Run Biotechnology Co., Ltd.
Berberine	CYR-X0024210628	≥ 98%	Sichuan Cuiyi Run Biotechnology Co., Ltd.

### 2.2 Determination of plant agronomic traits

Twenty-four fresh samples were measured for their plant height, number of leaves, leaf length, petiole length, fibrous root length, rhizome length, leaf width, rhizome diameter, and petiole diameter after being cleaned thoroughly.

### 2.3 Determination of total alkaloids content

Weigh 0.20 g of *C. chinensis* powder and introduce 50 mL of methanol-hydrochloric acid solution (100:1, v/v). The initial mass was recorded before an ultrasonic treatment (300 W, 80 kHz) for 30 minutes. After the total mass was restored to its original value by adding a methanol-hydrochloric acid solution, the mixture was centrifuged, and 1 mL of the supernatant was diluted to 100 mL in a volumetric flask. Measure the absorbance of the final solution at 270 nm.

Berberine was chosen as the evaluation indicator. A standard reference solution was prepared in methanol at a nominal concentration of 1 mg/mL and stored at 4°C until used. The content of berberine was calculated using the regression equations derived from calibration curves.

The evaluation of precision involved six consecutive analyses of the identical HSA1 solution, and the outcomes were presented as the relative standard deviation (RSD). Repeatability was evaluated by processing and analyzing six independent sample preparations of *C. chinensis* (HSA1) in parallel. Stability was determined by measuring the prepared HSA1 solution at designated intervals (0, 10, 20, 30, 40, and 50 minutes).

### 2.4 Determination of multi-component contents

Weigh 0.20 g accurately of *C. chinensis* powder and introduce 50 mL of methanol-hydrochloric acid solution (100:1, v/v). The initial mass was recorded before subjecting to ultrasonic treatment (300 W, 80 kHz) for 30 minutes. The total mass was restored to its original value by adding the methanol-hydrochloric acid solution after cooling to room temperature. The mixture was centrifuged, and 2 mL of the supernatant was diluted to 10 mL in a volumetric flask. Methanol was used to dilute to the mark, followed by thorough shaking and filtration to obtain the final sample solution. The sample solutions were filtered with a 0.45 μm syringe filter. The HPLC analyses were performed with an Agilent 1200 Series. Welch Xtimate^®^ C18 column (250 mm × 4.6 mm, 5 μm) was used. The solution (ammonium bicarbonate 2.37 g dissolved in 992 mL water, then added 7 mL ammonium hydroxide and 1 mL triethylamine, shaken well) was used as mobile phase A, methanol was used as mobile phase B, the gradient program as follows: 90% A ~ 75% A and 10% B ~ 25% B for 0 ~ 15 min, 75% A ~ 70% A and 25% B ~ 30% B for 15 ~ 25 min, 70% A ~ 55% A and 30% B ~ 45% B for 25 ~ 40 min, and 55% A ~ 90% A and 45% B ~ 10% B for 40 ~ 45 min. Injection volume, 10 μL; detection wavelength, 275 nm; column temperature, 30°C; and flow rate, 1.0 mL/min.

Epiberberine, coptisine, palmatine, berberine, magnoflorine, groenlandicine, jatrorrhizine, and columbamine were chosen as the evaluation indexes. Prepare the mixed reference solution of eight alkaloids in methanol at approximately 1 mg/mL and store it at 4°C until required for use. The concentrations of the eight target analytes were quantified using regression equations derived from their respective calibration curves.

The evaluation of precision involved six consecutive analyses of the identical HSA1 solution, and the outcomes were presented as the relative standard deviation (RSD). Repeatability was evaluated by processing and analyzing six independent sample preparations of *C. chinensis* (HSA1) in parallel. Stability was determined by measuring the prepared HSA1 solution at designated intervals (0, 2, 4, 8, 12, and 24 hours).

### 2.5 Non-targeted metabolomics analysis by liquid chromatography-mass spectrometry

To the weighed sample, add 600 µL methanol, containing 2-amino-3-(2-chlorophenyl) propanoic acid (4 mg/L). Homogenize by sonication at 55 Hz, then centrifuge at 1200 rpm and 4°C for 10 min. Transfer the supernatant through a 0.22 µm microporous filter membrane for LC-MS analysis (Vasilev et al., 2016).

### 2.6 Metagenomic sequencing

Construction of high-throughput sequencing libraries Macrogenomic sequencing was performed using the MGI T7 platform PE150 to obtain raw image data files of *C. chinensis* rhizome samples, which were converted into raw data (raw reads) through base calling analysis. Kraken2 software was used for macrogenomic species annotation based on reads. After filtering with fastp, effective sequencing data were obtained from the second-generation raw sequencing data. MEGAHIT software was used for metagenomic assembly of the second-generation data. minced software was used for CRISPR prediction of the genome; PhiSpy software was used to predict phages in the genome; IslandPath software was used to predict gene islands in the genome; functional annotation of target protein sequences was performed using multiple databases and alignment tools: GO annotations were assigned using BLASTP; COG annotations were generated with eggNOG-mapper employing DIAMOND BLASTP; ARDB, HI, CARD, VFDB, and CYPED annotations were conducted using BLASTP or DIAMOND BLASTP as appropriate; and CAZy database annotations were performed using HMMER; annotate the target protein sequence using diamond blastp based on the TCDB database; quantify the non-redundant gene set using the salmon software; perform differential analysis between paired samples using DESeq2 called by the trinityrnaseq software; perform statistical testing using the stats method in the scipy package in Python.

Microbial diversity was assessed by calculating α diversity indices and β diversity distances using the QIIME2 diversity module. Differential abundance analysis between paired samples was performed using both DESeq2 and edgeR implemented through the trinityrnaseq software.

### 2.7 Determination of physical and chemical properties of soils

The main physical and chemical properties of soil, including water content (WC), pH, Soil Organic Matter (SOM), Total Nitrogen (TN), Available Nitrogen (AN), Total Phosphorus (TP), Available Phosphorus (AP), Total Potassium (TK), Available Potassium (AK), urease (URE), sucrase (SUC), protease (PRO), catalase (CAT), were measured separately in accordance with the corresponding standard procedures.

### 2.8 Data analysis

Statistical analysis was performed using Excel 2016, with one-way analysis of variance (ANOVA) applied to determine significance. Results with *P* < 0.05 were considered statistically significant, and data are expressed as “mean ± standard deviation”. Figures were generated using GraphPad Prism 8.0 and arranged with Adobe Illustrator 2020.

## 3 Results

### 3.1 The effect of altitude on the agronomic traits of *C. chinensis* plants

**Figure 1:**
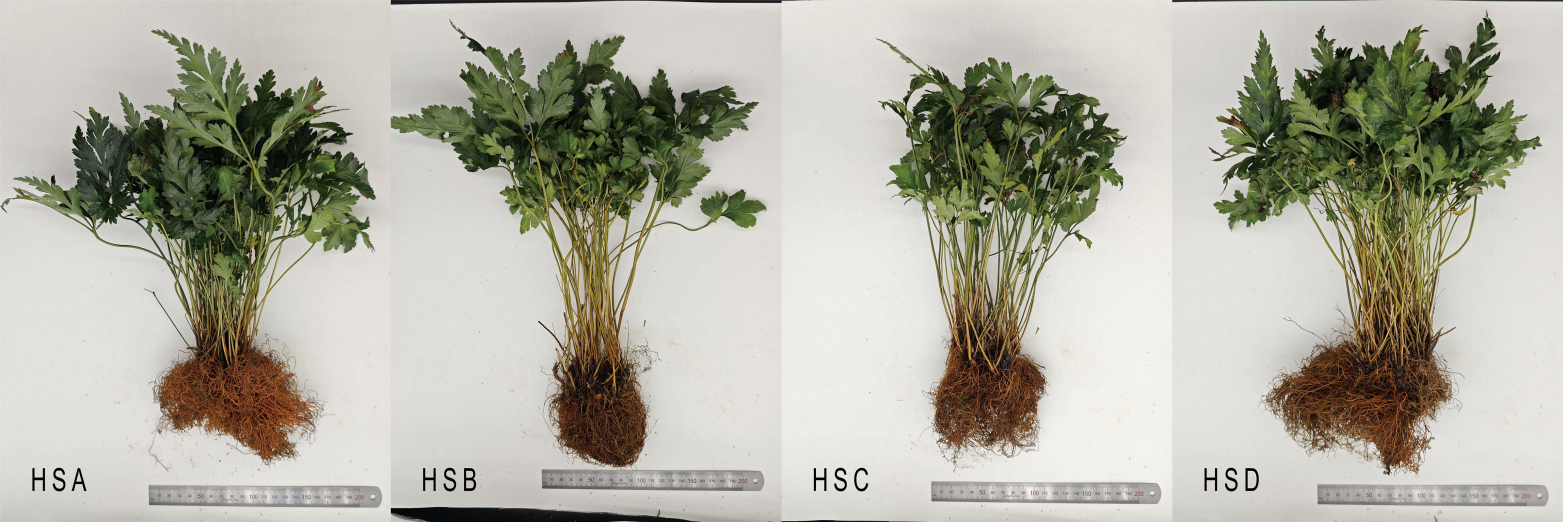
C*. chinensis* plants at different altitudes.

The growth of *C. chinensis* exhibited significant variation across different altitudinal gradients (shown in **Figure 1**, **Table 5**). Significant morphological variations in *C. chinensis* were observed across the four altitudinal gradients. Branch number, plant height, leaf dimensions (width and length), and petiole characteristics (length and diameter) exhibited highly significant differences (*P* < 0.01). Rhizome diameter also showed a significant altitudinal variation (*P* < 0.05). In contrast, fibrous root length and rhizome length did not demonstrate significant differences across the elevation gradients.

**Table 5:** Agronomic traits of *C. chinensis* plants at different altitudes (*n* = 6).

Group	Number of branches	Plant height	Leaf width	Leaf length	Petiole length	Petiole diameter	Fibrous root length	Rhizome length	Rhizome diameter
Measurement units	pcs	cm	cm	cm	cm	mm	cm	cm	cm
HSA	29 ± 6.98A	32.63 ± 1.77A	6.28 ± 0.74B	12.28 ± 1.64C	16.45 ± 4.44A	1.70 ± 0.19AB	10.85 ± 2.23	7.13 ± 1.28	8.60 ± 2.53a
HSB	22 ± 3.54A	32.72 ± 1.74BC	6.13 ± 1.00B	11.95 ± 2.05ABC	15.37 ± 4.48AB	1.55 ± 0.20AB	10.85 ± 2.23	6.63 ± 0.94	6.97 ± 2.44b
HSC	51 ± 16.08B	30.90 ± 5.14B	5.82 ± 1.31B	11.62 ± 2.11A	14.62 ± 4.12B	1.58 ± 0.25A	11.73 ± 1.46	6.72 ± 0.95	6.47 ± 2.89b
HSD	57 ± 8.29B	30.22 ± 4.93BC	5.87 ± 1.35A	12.12 ± 2.41BC	14.42 ± 3.84AB	1.64 ± 0.28C	12.38 ± 1.09	5.98 ± 1.08	6.35 ± 2.86ab

In the table, different capital letters indicate extremely significant differences (*P* < 0.01), and different lowercase letters indicate significant differences (*P* < 0.05), the same as below.

The size of the medicinal rhizomes showed a decreasing trend with increasing altitude, suggesting that high altitudes may inhibit the growth of medicinal parts. Conversely, fibrous root length tended to increase with altitude, indicating that high-altitude environments may be more conducive to the growth of fibrous roots in *C. chinensis*.

### 3.2 The effect of altitude on the content of active ingredients in *C. chinensis*

**Figure 2:**
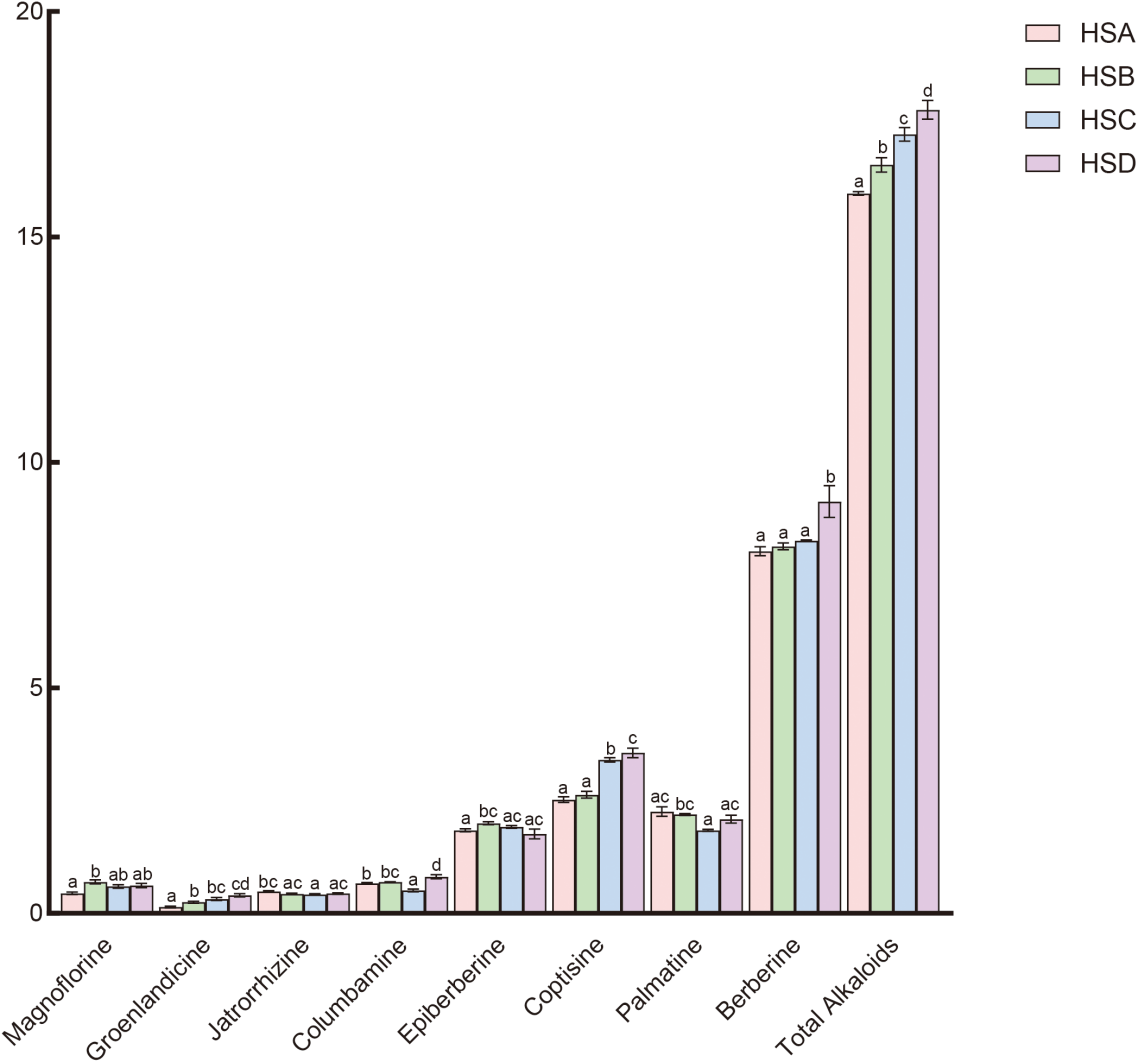
Effects of altitude on the content accumulation of active components in *C. Chinensis.* Different letters indicate significant differences, ANOVA *P* < 0.05.

With the increase in cultivation altitude, the contents of groenlandicine, coptisine, berberine, and total alkaloids in *C. chinensis* show a certain upward trend, and the total accumulation of active components in high-altitude areas is higher than that in low-altitude areas (**Figure 2**). However, the contents of magnoflorine, jatrorrhizine, columbamine, epiberberine, and palmatine hydrochloride do not show a regular trend related to altitude changes. Among them, the total alkaloid content increases from 15.97% at 907 m to 17.82% at 1698 m (*P* < 0.01).

### 3.3 Analysis of metabolic products in rhizomes of *C. chinensis* at different altitudes

**Figure 3:**
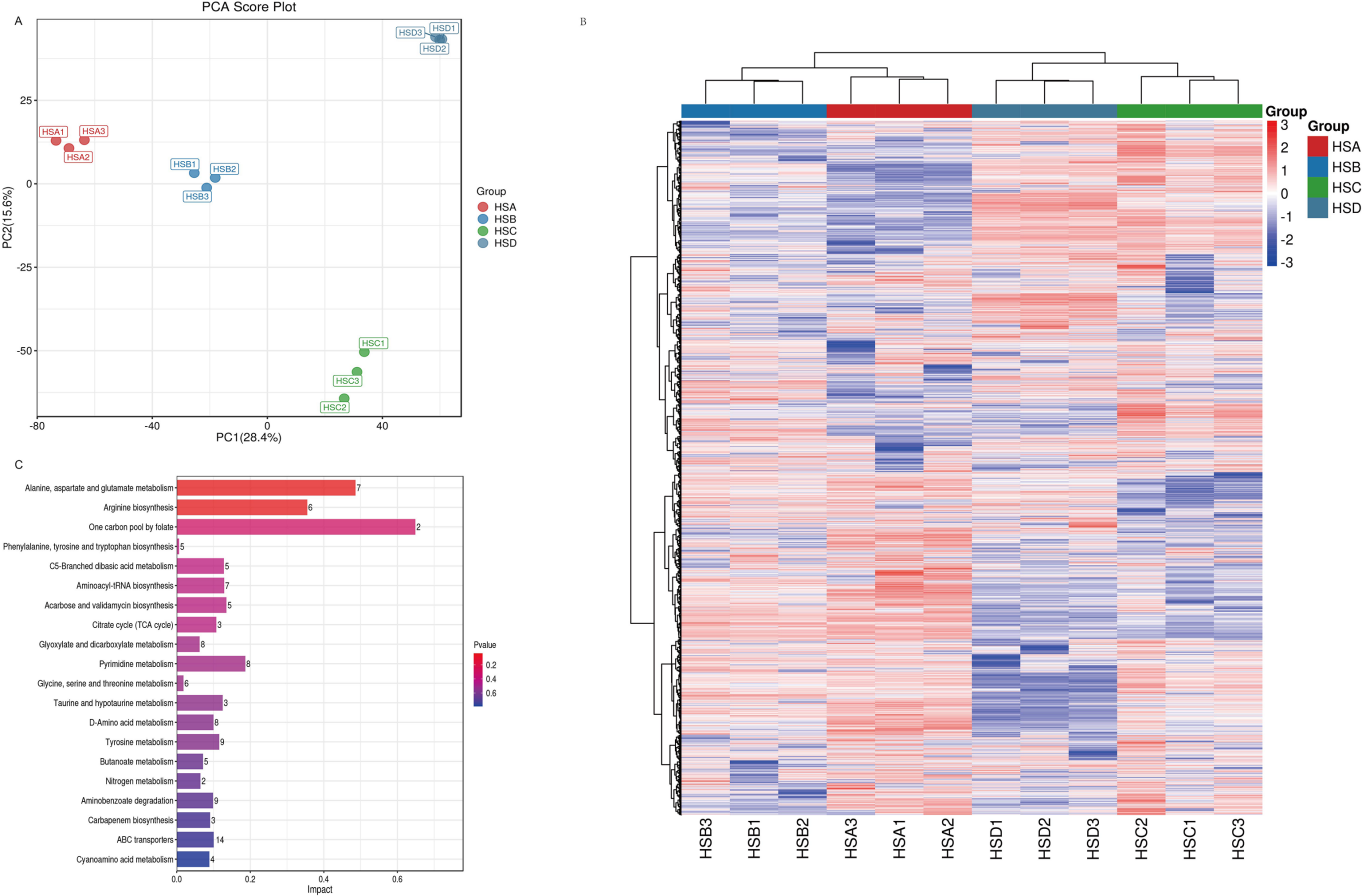
Metabolomics analysis of rhizomes of *Coptis chinensis* at different altitudes. **(A)** PCA; **(B)** KEGG; **(C)** Heatmaps of the DEMs compared with different altitudes.

Metabolomics analysis was conducted on the rhizomes of *C. chinensis* collected at different altitudes. Principal component analysis (PCA) revealed distinct clustering of replicate samples within each group, with the model explaining 54.7% of the total variance (**Figure 3A**). A total of 912 differentially expressed metabolites (DEMs) were identified in the comparisons among HSA, HSB, HSC, and HSD. Among these, the dominant categories were alkaloids and derivatives (29 compounds, accounting for 37.28% ~ 52.46%), organic acids and derivatives (79 compounds, accounting for 8.89% ~ 16.64%), phenylpropanoids and polyketides (137 compounds, accounting for 8.67% ~ 12.83%), and organoheterocyclic compounds (124 compounds, accounting for 4.97% ~ 6.78%), collectively accounting for 71.76% ~ 80.41% of all DEMs. KEGG pathway enrichment analysis revealed that these differential metabolites were predominantly enriched in amino acid metabolism pathways, particularly alanine, aspartate, glutamate, and arginine biosynthesis, as well as biosynthetic pathways including tyrosine and pyrimidine metabolism. Additionally, pathways related to genetic information transmission (e.g., aminoacyl-tRNA biosynthesis) and substance transmembrane transport (e.g., ABC transporter pathways) were also significantly enriched (**Figure 3B**). Heatmap results demonstrated distinct accumulation patterns of metabolites in *C. chinensis* rhizomes across the four altitudes, with differentially expressed metabolites showing clear variations in their accumulation trends at different altitudes (**Figure 3C**).

### 3.4 Sequencing analysis of rhizomes of *C. chinensis* microorganisms at different altitudes

#### 3.4.1 Analysis of sequencing depth of *C. chinensis* rhizomes at different altitudes

**Figure 4:**
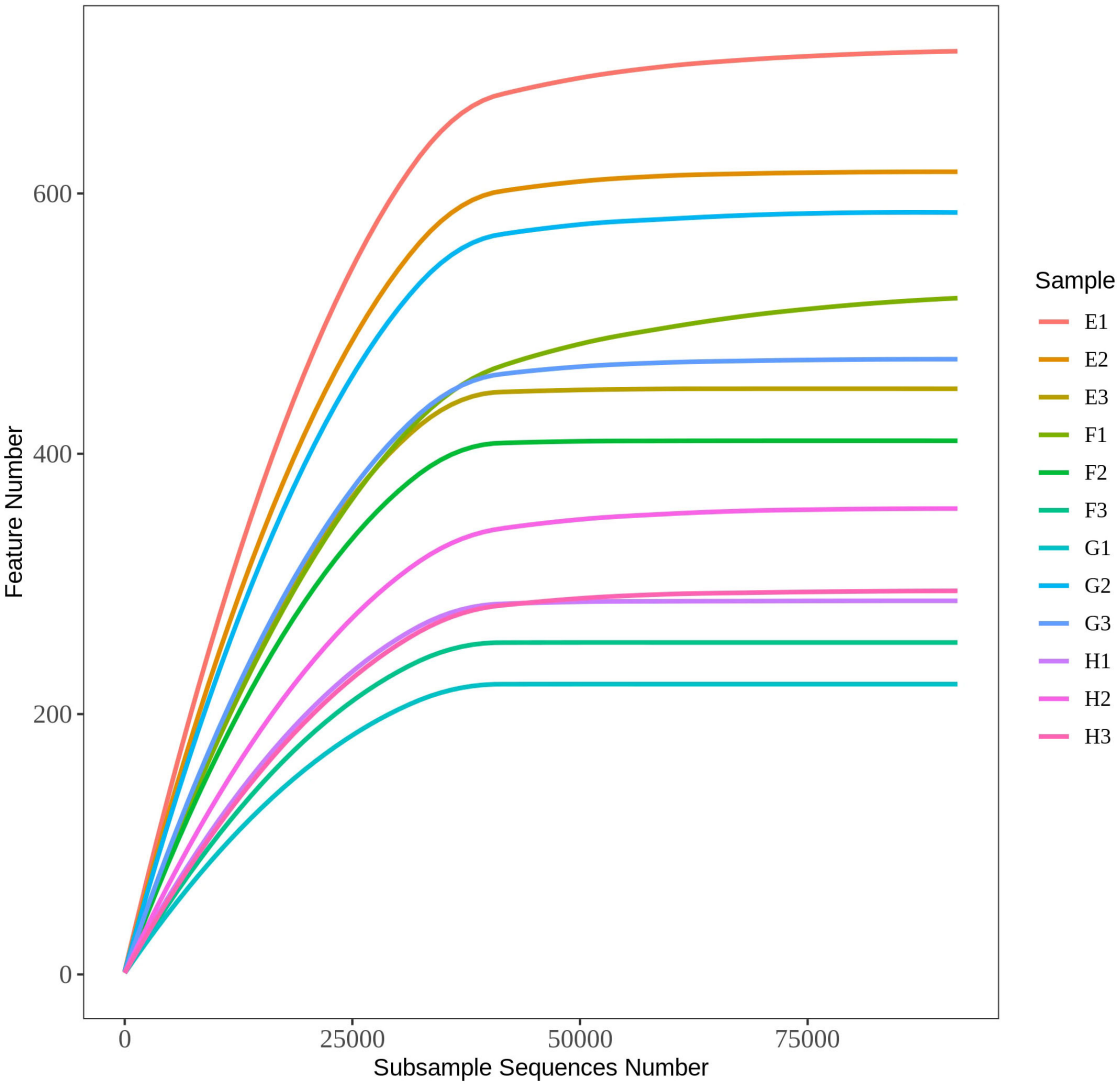
Rarefaction curves of rhizome of *C. chinensis* at different altitudes E (E1, E2, and E3) are HSA plant samples; F (F1, F2, and F3) are HSB plant samples; G (G1, G2, and G3) are HSC plant samples; H (H1, H2, and H3) are HSD plant samples, the same below.

Rarefaction curves can indicate whether the sequencing depth can cover all sequencing targets in the test samples and the rationality of the sequencing results. As shown in **Figure 4**, when the sequencing quantity reaches 40000, the curve gradually becomes parallel, indicating that the sequencing data volume can more comprehensively reflect the microbial community composition of the sequenced samples.

#### 3.4.2 α-diversity analysis of rhizomes of *C. chinensis* at different altitudes

**Figure 5:**
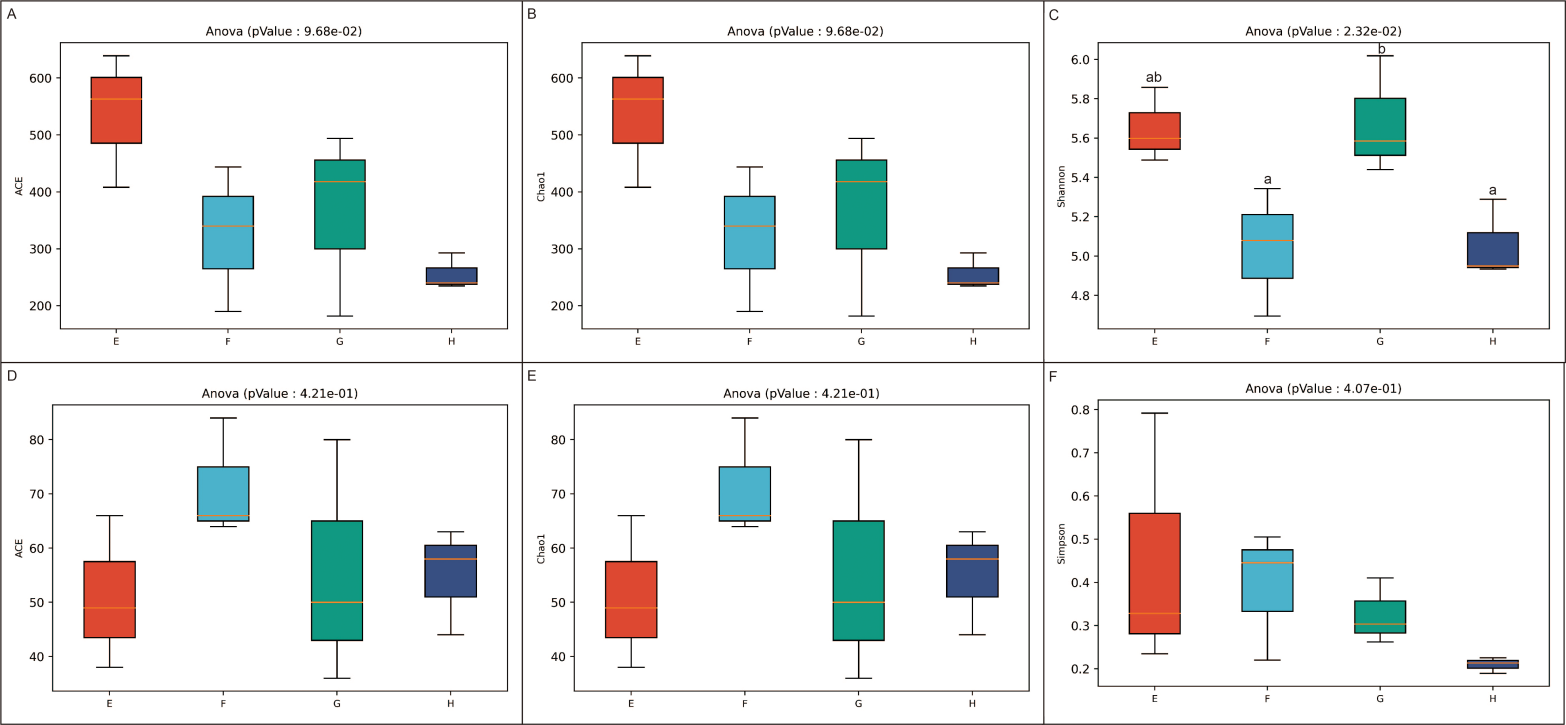
Alpha diversity indices of rhizome communities of *C. chinensis* at different altitudes. Different lowercase letters indicate significant differences, ANOVA *P* < 0.05. **(A–C)** correspond to the ACE index, Chao 1 index, and Simpson index of endophytic bacteria in rhizomes, respectively; **(D–F)** correspond to the ACE index, Chao 1 index, and Simpson index of endophytic fungi in rhizomes, respectively.

For the endophytic microbial communities in the rhizomes of *C. chinensis*, α-diversity analysis reflects key metrics of species richness and evenness: a larger ACE index and Chao 1 index indicate higher species richness of microbes in the community, while a smaller Simpson index indicates greater species diversity of the community. As shown in **Figure 5**, the Simpson index of endophytic bacteria differs significantly across altitudes among different groups (*P* < 0.05), while no significant differences are observed for the other indices. Meanwhile, the species richness of fungal communities remains comparable across groups. Synthesizing the analysis results of the diversity indices of both bacterial and fungal endophytes, it is indicated that the species richness and evenness of endophytic microbial communities at different altitudes exhibit similarity, and the number of species within the communities as well as the distribution uniformity of dominant species were not significantly affected by altitude.

#### 3.4.3 β-diversity analysis of rhizome microorganisms of *C. chinensis* at different altitudes

**Figure 6:**
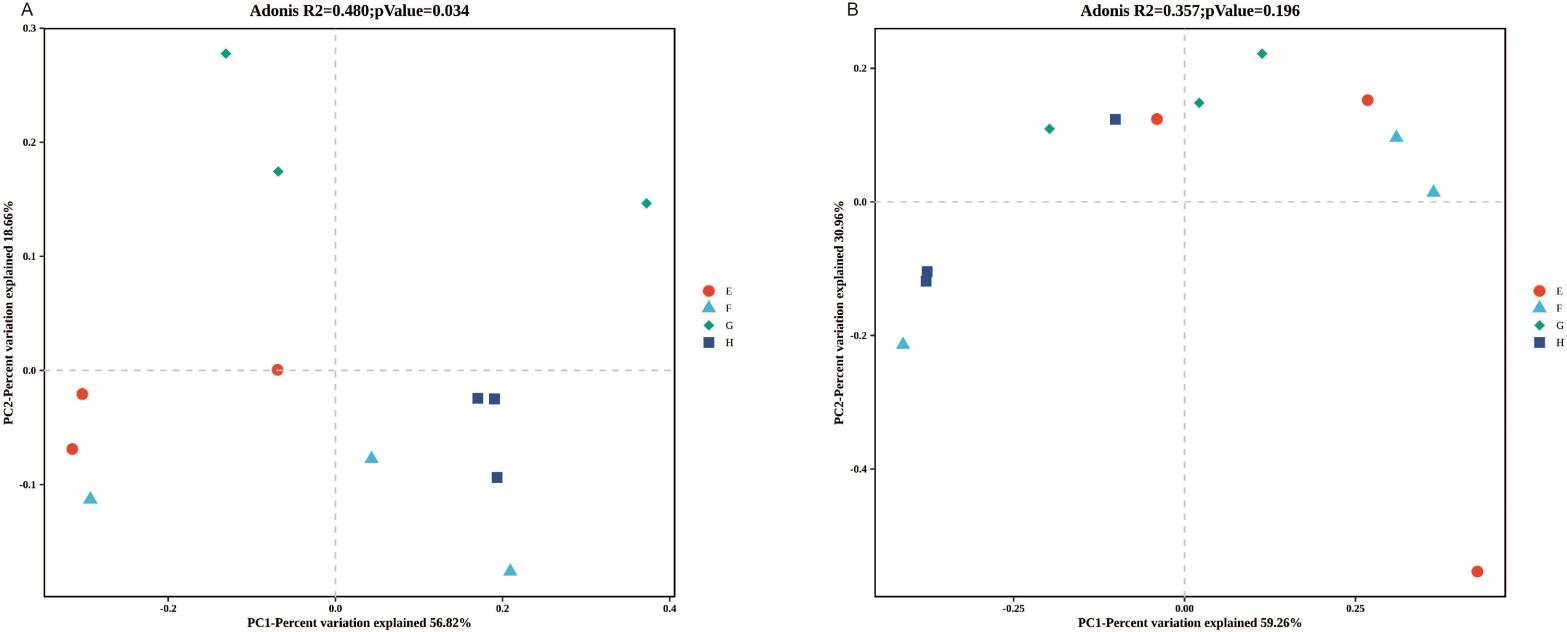
PCoA sequencing analysis based on bray curtis Algorithm. **(A)** bacteria. **(B)** fungi.

To assess the influence of altitudinal variation on rhizome microbial composition, principal coordinate analysis (PCoA) was applied using Bray-Curtis dissimilarity metrics. It was shown that the bacterial community composition varied among the four altitudes, while no significant difference was observed in fungal community composition. For bacteria, PC1 and PC2 collectively explained 75.49% of the community variation (PC1: 56.83%, PC2: 18.66%); for fungi, PC1 and PC2 collectively explained 89.72% of the community variation (PC1: 59.26%, PC2: 30.46%), which could effectively reflect the difference pattern of microbial community composition among samples (**Figure 6**). The explanatory power of different altitudes for bacterial community structure was 48% (*P* = 0.034), which indicates that the effect of altitude on the endophytes of *C. chinensis* is not achieved by altering their richness, but rather by regulating the community composition and structure of the endophytes in *C. chinensis*.

#### 3.4.4 Analysis of microbial community characteristics of *C. chinensis* rhizomes at different altitudes

**Figure 7:**
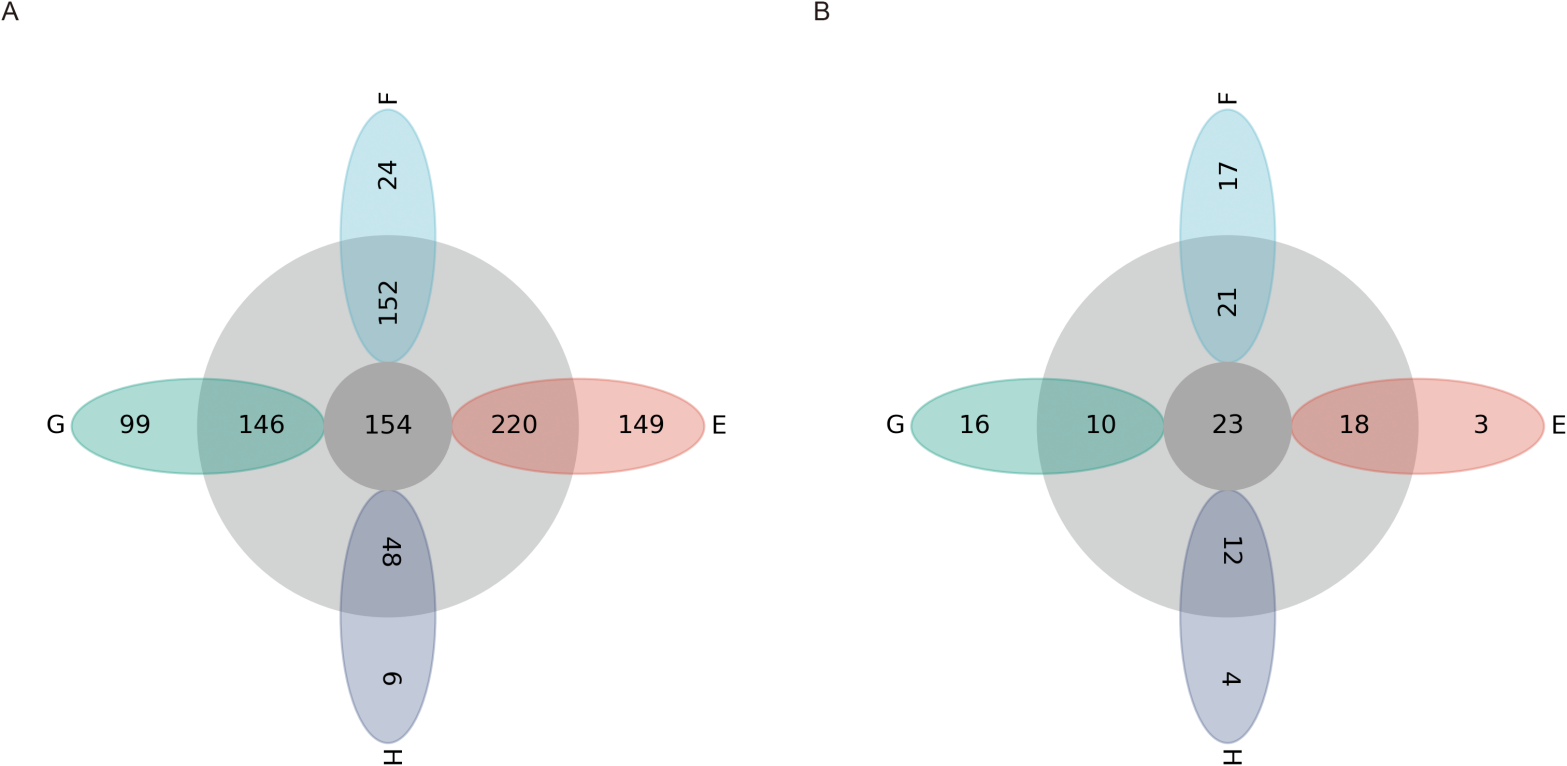
Petal diagrams of microbial communities in rhizomes of *C chinensis* at different altitudes. **(A)** bacteria. **(B)** fungi.

After comparative identification of the representative sequences of microbial OTUs in the rhizomes of *C. chinensis* at different altitudes, 25 phyla, 46 classes, 92 orders, 164 families, 333 genera, and 760 species were obtained. As shown in **Figure 7A**, the number of unique bacteria at an altitude of 907 m was the largest, with 149, and there were 523 OTUs at 907 m; the number of unique microorganisms at 1697 m was the smallest, with 6, and there were 208 OTUs at 1697 m. The composition of dominant fungal communities in the rhizomes of *C. chinensis* at different altitudes was similar, with no significant differences (**Figure 7B**).

**Figure 8:**
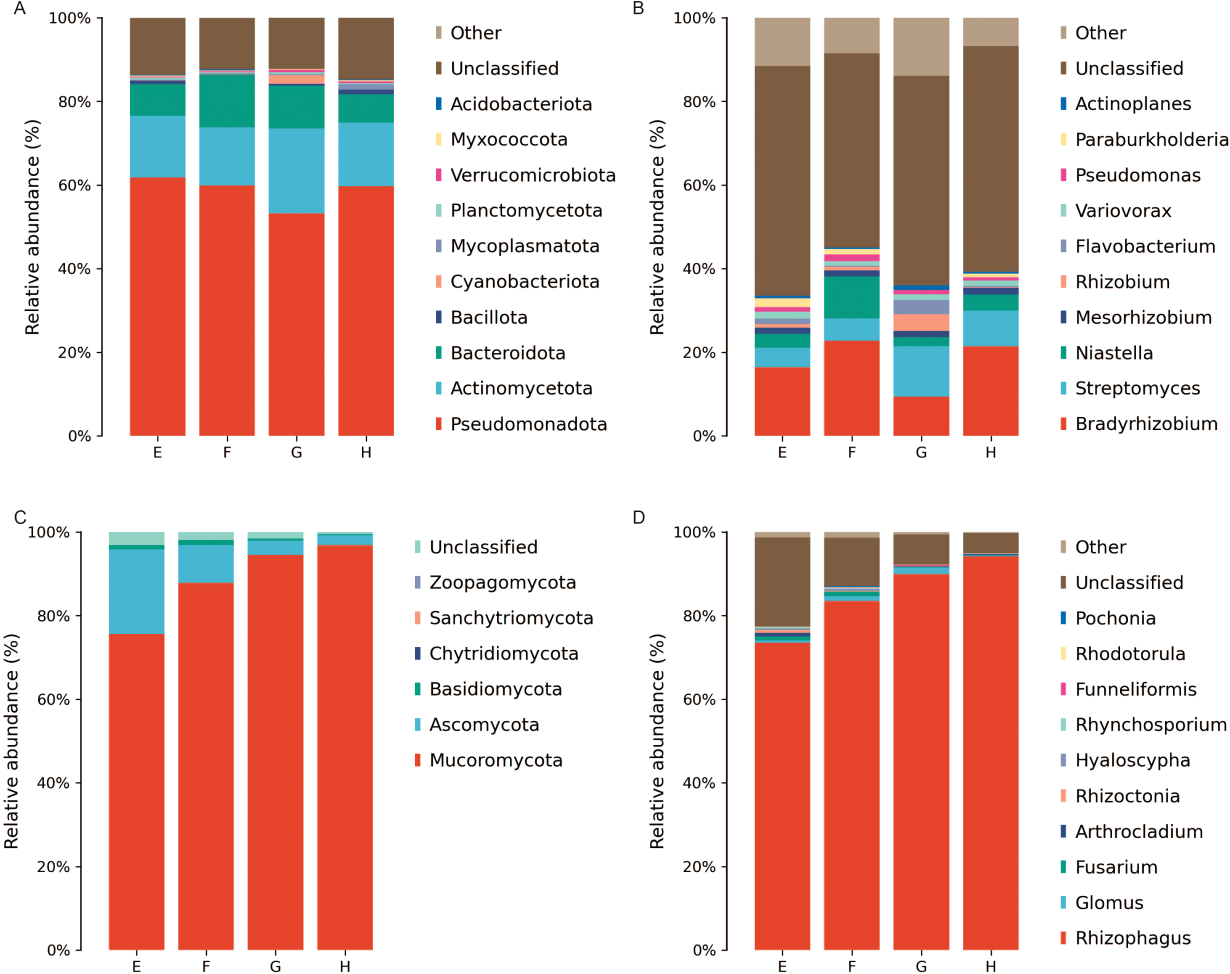
Composition of rhizome microbial communities of *C chinensis* at different altitudes (at the phylum and genus levels). **(A)** Phylum level of bacteria. **(B)** Genus level of bacteria. **(C)** Phylum level of fungi. **(D)** Genus level of fungi.

The dominant bacterial phyla in the rhizomes of *C. chinensis* across different altitudes, at the phylum level, were Pseudomonadota (53.17% ~ 61.81%), Actinomycetota (13.91% ~ 20.35%), Bacteroidota (6.80% ~ 12.55%), Bacillota (0.14% ~ 1.07%), Cyanobacteriota (0.02% ~ 2.08%), Mycoplasmatota (0.10% ~ 1.32%), Planctomycetota (0.27% ~ 0.58%), Verrucomicrobiota (0.18% ~ 0.58%), Myxococcota (0.17% ~ 0.90%), and Acidobacteriota (0.02% ~ 0.22%) (**Figure 8A**). At the genus level, the dominant bacterial genera in *C. chinensis* rhizomes across different altitudes were *Bradyrhizobium*, *Streptomyces*, and *Niastella*, in order of abundance, with the proportions of all other bacterial taxa being less than 5% (**Figure 8B**).

The main fungal phyla in *C. chinensis* rhizomes across different altitudes, at the phylum level, were Mucoromycota (75.58% ~ 96.90%), Ascomycota (2.29% ~ 20.27%), and Basidiomycota (0.25% ~ 1.05%) (**Figure 8C**). Among these, the relative abundances of Mucoromycota and Ascomycota were notably influenced by altitude: the former exhibited an increasing trend with rising altitude, while the latter showed a decreasing trend. At the genus level, the dominant fungal genus in *C. chinensis* rhizomes across different altitudes was *Rhizophagus*, with the proportions of all other fungal taxa being less than 5% (**Figure 8D**). Additionally, the relative abundance of *Rhizophagus* was significantly affected by altitude, exhibiting a marked increasing trend with rising altitude.

#### 3.4.5 Metagenomic functional annotation reveals the metabolic potential of endophytic communities in *C. chinensis* rhizomes

To elucidate the functional characteristics of the endophytic microbial community in *C. chinensis* rhizomes, we performed KEGG pathway annotation analysis on the metagenomic data of endophytes from *C. chinensis* at different altitudes. The KEGG annotation results for bacteria (**Supplementary Figure S1**) show that their gene functions are widely distributed across major pathway categories. The pathways with the most prominent number of annotated genes are concentrated under the “Metabolism” category. Among these, amino acid metabolism-related pathways were heavily annotated, such as “Alanine, aspartate and glutamate metabolism” and “Glycine, serine and threonine metabolism”. The number of annotated genes for these pathways remains at an extremely high level across all altitude groups, highlighting the endophytic bacterial community’s active and robust capacity for amino acid synthesis and conversion, which provides pathway support for the synthesis of precursor substances of *C. chinensis* alkaloids. Furthermore, a considerable number of genes are also annotated in other major categories, including “Cellular Processes”, “Environmental Information Processing”, and “Genetic Information Processing”, indicating that the bacterial community also plays important roles in maintaining basic cellular activities, adapting to the environment, and facilitating the transmission of genetic information flow. The overview of KEGG functional annotation for endophytic fungi is shown in **Supplementary Figure S2**. Similar to bacteria, fungi possess a wealth of functional genes related to core metabolism, secondary metabolism, and environmental interactions.

### 3.5 The changes of soil physicochemical properties of *C. chinensis* at different altitudes

The pH was slightly acidic (ranging from 4.61 to 5.83), and the altitude of the HSA group was significantly lower than that of the other sampling sites. The water content (WC), total nitrogen (TN), available nitrogen (AN), available potassium (AK), and soil organic matter (SOM) in high-altitude areas were significantly higher than those in low-altitude areas, but they did not show an increasing trend with increasing altitude. However, unlike AK, total potassium (TK) showed a decreasing trend with increasing altitude. The total phosphorus (TP) content ranged from 1.09 to 2.29 g/kg, with significant differences among samples (*P* < 0.01): the HSA sample had the highest TP content, while the HSC sample had the lowest. The available phosphorus (AP) content varied highly significantly among samples (*P* < 0.01), and the AP content of the HSA sample was significantly higher than that of the other samples (shown in **Table 6**).

**Table 6:** Soil nutrients of *C. chinensis* at different altitudes.

Sample	pH	TN (g/kg)	SOM (g/kg)	TP (g/kg)	TK (g/kg)	AN (mg/kg)	AP (mg/kg)	AK (mg/kg)	WC(%)
HSA	4.61 ± 0.01a	3.12 ± 0.06ab	60.17 ± 1.68B	2.29 ± 0.05D	26.66 ± 0.54c	203.16 ± 1.04B	549.00 ± 8.72D	468.19 ± 4.50B	24.64 ± 0.80A
HSB	5.43 ± 0.01ab	2.23 ± 0.03a	44.35 ± 0.88A	1.52 ± 0.08B	26.48 ± 0.57c	132.35 ± 0.68A	249.13 ± 2.82C	407.02 ± 5.73A	23.02 ± 1.33A
HSC	5.06 ± 0.02ab	4.27 ± 0.05ab	85.78 ± 1.04C	1.09 ± 0.03A	25.20 ± 0.42b	318.77 ± 1.04C	75.96 ± 0.38A	513.86 ± 5.15D	34.74 ± 1.76C
HSD	5.83 ± 0.02b	4.91 ± 0.09b	93.12 ± 0.96D	2.03 ± 0.01C	14.49 ± 0.14a	319.68 ± 0.68C	165.29 ± 3.36B	497.65 ± 3.96C	30.28 ± 1.38D

The urease (URE) activity ranged from 627.86 to 1026.61 U/g, and the URE activity showed an increasing trend with increasing altitude. The increase in urease activity may facilitate the conversion of soil organic nitrogen to available nitrogen (Kuscu, 2019), providing nitrogen nutrition for the growth of *C.* chinensis. The invertase (SUC) activity ranged from 599.16 to 1239.77 U/g, and the HSC sample had the highest SUC activity. The catalase (CAT) activity ranged from 26.00 to 31.77 U/g, and the HSA sample had the lowest CAT activity, which was significantly lower than that of the other three samples (*P* < 0.01). The acid protease (PRO) activity ranged from 2.78 to 4.40 U/g, with significant differences among samples: the HSD sample had the highest PRO activity, which was significantly higher than that of the other three samples (*P* < 0.01) (shown in **Table 7**).

**Table 7:** Soil enzyme activities of *C. chinensis* at different altitudes.

Sample	URE(U/g)	SUC(U/g)	CAT(U/g)	PRO(U/g)
HSA	627.86 ± 26.63a	935.89 ± 41.63a	26.00 ± 1.45A	3.22 ± 0.29A
HSB	823.04 ± 14.63b	599.16 ± 30.65ab	31.65 ± 1.07B	2.78 ± 0.19A
HSC	785.78 ± 12.59c	1239.77 ± 135.71ab	31.77 ± 0.32B	3.12 ± 0.30A
HSD	1026.61 ± 13.72d	1032.65 ± 38.08b	29.59 ± 0.48B	4.40 ± 0.09B

### 3.6 Correlation analysis between the microecology and quality of *C. chinensis* at different altitudes

**Figure 9:**
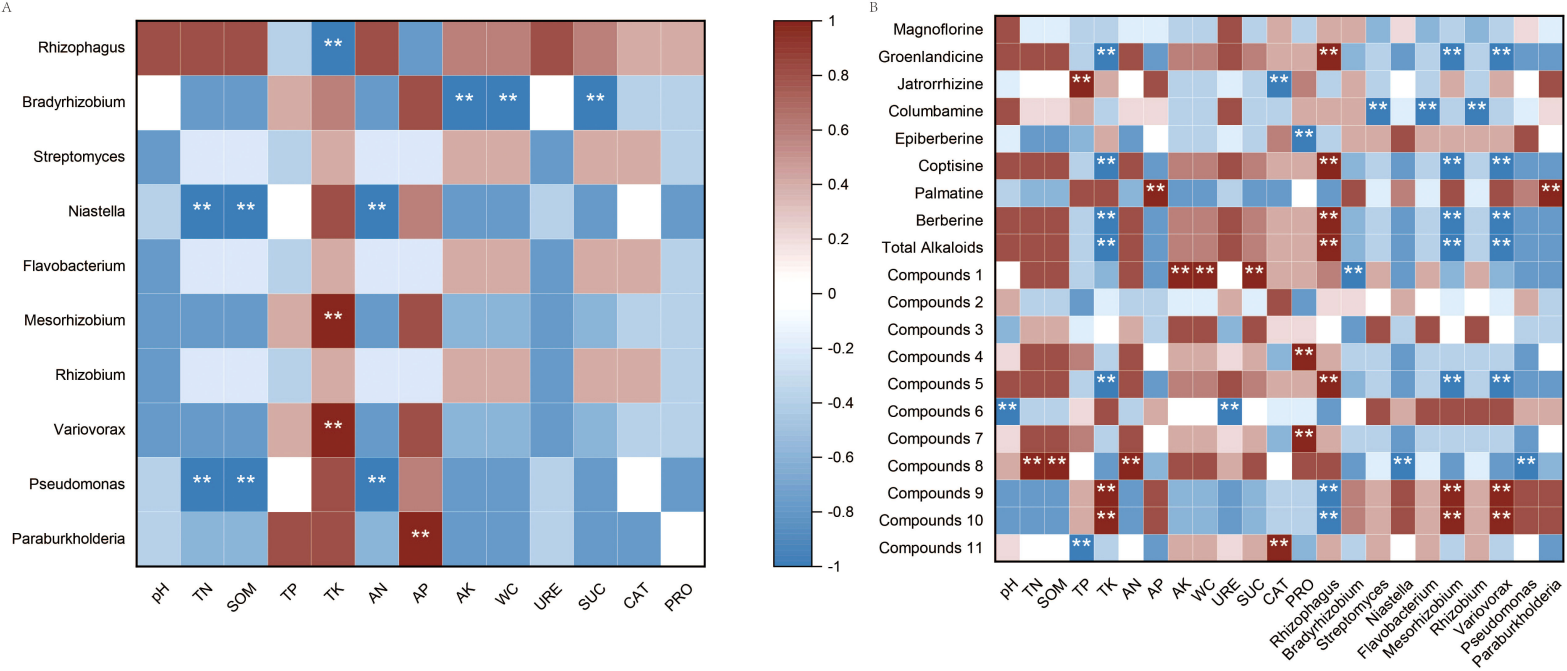
Spearman correlation heatmap. **(A)** Spearman correlation analysis between microorganisms in rhizomes and physical and chemical properties of soils at different altitudes (at the genus level); **(B)** Spearman correlation analysis among microorganisms in rhizomes (at the genus level), physical and chemical properties of soils, and active components at different altitudes. “**” indicates that the correlation reaches an extremely significant level (*P* < 0.01); the correlation coefficient ranges from -1 to 1, where the range from -1 to 0 represents a negative correlation, and the range from 0 to 1 represents a positive correlation. Compounds 1: Organic nitrogen compounds; Compounds 2: Organosulfur compounds; Compounds 3: Organic oxygen compounds; Compounds 4: Benzenoids; Compounds 5: Lipids and lipid-like molecules; Compounds 6: Lignans, neolignans and related compounds; Compounds 7: Nucleosides, nucleotides, and analogues; Compounds 8: Organoheterocyclic compounds; Compounds 9: Phenylpropanoids and polyketides; Compounds 10: Organic acids and derivatives; Compounds 11: Alkaloids and derivatives.

The top 10 dominant microbial genera in rhizomes based on relative abundance were selected, and Spearman correlation analysis was employed to evaluate associations among these microbial genera, soil physicochemical properties, and the relative abundance of differentially expressed metabolites (DEMs). The results showed that *Bradyrhizobium* exhibited extremely significant positive correlations with total nitrogen (TN) and available nitrogen (AN), while showing an extremely significant negative correlation with total potassium (TK); *Niastella* was extremely significantly positively correlated with soil organic matter (SOM) and urease (URE) activity; *Streptomyces* presented an extremely significant negative correlation with available phosphorus (AP); *Pseudomonas* showed extremely significant positive correlations with TN and AN (visualized in **Figure 9A**).

As visualized in **Figure 9B**, organic sulfur compounds, phenols, lignans and neolignans, nucleosides, organic heterocyclic compounds, phenylpropanoids, polyketides, organic acids and their derivatives were all related to the distribution of microbial communities in *C. chinensis*. Associations between specific metabolite classes and microbial genera were identified through correlation analysis. Organic sulfur compounds exhibited a significant positive correlation with *Niastella* but a negative correlation with *Mesorhizobium*. In contrast, lignans, neolignans, phenylpropanoids, polyketides, and organic acids and derivatives were positively correlated with both *Niastella* and *Mesorhizobium*; organic heterocyclic compounds showed significant negative correlations with these two genera. Benzenoids demonstrated a positive correlation with *Rhizophagus* and negative correlations with *Streptomyces* and *Flavobacterium*. Lastly, nucleosides were negatively correlated with *Variovorax*. Sucrase activity, as well as the contents of groenlandicine, columbamine, coptisine, palmatine hydrochloride, berberine hydrochloride, and total alkaloids, were related to the distribution of microbial communities in the rhizomes of *C. chinensis*. The contents of groenlandicine, coptisine, berberine hydrochloride, and total alkaloids were strongly positively correlated with *Rhizophagus*, and significantly negatively correlated with *Mesorhizobium* and *Variovorax*; columbamine showed a strongly negative correlation with *Streptomyces*, *Flavobacterium*, and *Rhizobium*; palmatine hydrochloride was positively correlated with *Paraburkholderia*. Metabolites of *C. chinensis* were not only associated with the distribution of microbial communities but also showed significant correlations with key soil physicochemical properties. SOM was extremely significantly positively correlated with lignans, neolignans, phenylpropanoids and polyketides, while pH was extremely significantly negatively correlated with groenlandicine and coptisine; AP was extremely significantly positively correlated with organosulfur compounds.

## 4 Discussion

### 4.1 Altitude drives the specific differentiation of the endophytic microbial community and the enrichment of beneficial bacteria in *C. chinensis* rhizomes

Environmental factors such as temperature and precipitation exert a greater impact on the diversity of endophytes in leaves and stems (Cheng et al., 2024). Plant microecological communities construct specifically based on the ecosystem characteristics of different altitudes, and their diversity shows significant differences across altitudes (Reyes Ardila et al., 2024). Endophytes exhibit varying degrees of plant growth-promoting, colonization, and pathogen-resistant properties (Bashir et al., 2023). In this study, the dominant microbial phyla (Mucoromycota, Pseudomonadota, Actinobacteriota) in the rhizomes of *C. chinensis* from the Shizhu area are consistent with the dominant taxa reported in previous studies on medicinal plants (Alami et al., 2020; Liao et al., 2021), and the endophytic community of *C. chinensis* exhibits a unique altitude adaptation pattern.

Environmental filtering along the altitude gradient has promoted the enrichment of beneficial endophytic genera with specific functions in the rhizomes of *C. chinensis*, such as *Bradyrhizobium*, *Rhizophagus*, and *Paraburkholderia*. Among these, *Bradyrhizobium*, as a typical nitrogen-fixing microorganism, can convert atmospheric nitrogen into plant-available ammonium nitrogen, supplement the soil nitrogen pool, and enhance nitrogen supply capacity. Its positive correlation with soil nitrogen content may stem from the sufficient nitrogen providing the necessary nutritional basis for the reproduction and colonization of this genus. Meanwhile, *Rhizophagus*, as a typical arbuscular mycorrhizal fungus (AMF), showed no significant correlation between rhizosphere soil pH and its relative abundance in this study. However, previous studies have indicated that low pH inhibits its growth and functions (Wang et al., 2017), which may be one of the important reasons for the relatively low abundance of *Rhizophagus* in the low-altitude group (HSA).

### 4.2 Altitude regulates metabolic pathways and precursor supply, thereby promoting the synthesis of secondary metabolites in *C. chinensis*

It is confirmed that altitude-related environmental differences can alter plant alkaloid accumulation patterns by affecting the transcriptional activity of alkaloid biosynthetic genes, enzymatic reaction efficiency, and other factors (Lee et al., 2020; Koirala et al., 2023; Liu et al., 2023b). This study reveals the specific manifestations of this regulatory mechanism in *C. chinensis*. As 5altitude increases, with lower temperatures and more scarce nutrient conditions, plants are prompted to enhance the synthesis of defensive secondary metabolites, thereby improving their adaptability to extreme environments (Ma et al., 2021). As the core precursor for the synthesis of benzylisoquinoline alkaloids (BIAs), tyrosine’s enhanced metabolic flux directly provides sufficient substrates for the production of (S)-reticuline, thereby promoting the efficient synthesis of target alkaloids such as groenlandicine and berberine (He et al., 2018; Xu et al., 2024).

The differentially expressed metabolites (DEMs) across different altitudes are mainly involved in pathways such as tyrosine metabolism, which is one of the reasons for the increase in alkaloid content with rising altitude. The biosynthesis of benzylisoquinoline alkaloids (BIAs) initiates with the decarboxylation and transamination of tyrosine, a process highly dependent on nitrogen availability. As a fundamental nutrient for plant growth and metabolism, nitrogen supply directly influences the direction of alkaloid synthesis (Lee and Facchini, 2011). Previous studies have shown that *Mesorhizobium* carrying the nifH gene is a key nitrogen cycle-functional bacterium (Haukka et al., 1998; Lewin et al., 2024), capable of fixing atmospheric N_2_ through symbiosis with plants (Haukka et al., 1998). However, under conditions of sufficient nitrogen supply, plants may preferentially utilize tyrosine for protein synthesis (e.g., enzymes, structural proteins) rather than secondary metabolites such as alkaloids (Park et al., 2021). This indicates that altitude can indirectly regulate the substrate allocation direction of alkaloid synthesis in *C. chinensis* by modulating soil nitrogen levels and the activity of related functional bacteria.

### 4.3 Interaction mechanism between endophytic communities and metabolite accumulation in *C. chinensis*

#### 4.3.1 Regulatory roles of endophytes in alkaloid biosynthesis

Elucidating the interactions among medicinal plants, endophytes, and secondary metabolites is essential for enhancing plant growth, improving quality, and mitigating continuous cropping obstacles (Wu et al., 2021; Singh et al., 2022). Dominant endophytic genera such as *Bradyrhizobium*, *Rhizophagus*, and *Paraburkholderia* can significantly inhibit the growth and reproduction of plant pathogens through multiple mechanisms including nutrient and niche competition, secretion of antimicrobial substances, and induction of systemic resistance (Wang et al., 2025), thereby maintaining the endophytic microbial balance in *C. chinensis* rhizomes and enhancing its disease resistance to promote growth. In addition, these dominant species are involved in alkaloid synthesis through direct or indirect effects (Liu et al., 2020; Cao et al., 2025).

*Rhizophagus* can form a symbiotic relationship with *C. chinensis*. By activating AMF-plant symbiotic signaling to induce the expression of related genes, it regulates the expression of ammonium transporter LeAMT1.1 and nitrate transporter LeNRT2.3 to optimize nitrogen utilization (Xie et al., 2023b). Based on the benzylisoquinoline alkaloids (BIAs) biosynthesis pathway, it is speculated that this enhanced nitrogen supply can directly increase the substrates required for alkaloid synthesis (Min et al., 2023). There have been relevant reports on *Rhizophagus*-mediated metabolite accumulation in medicinal plants. In *Glycyrrhiza uralensis*, *Rhizophagus irregularis* not only induces the accumulation of specific phenols and flavonoids under drought stress to enhance drought resistance, but also upregulates the expression of squalene synthase 1 (a key enzyme for glycyrrhizin synthesis) and chalcone synthase (a key enzyme for flavonoid synthesis) when co-inoculated with *Trichoderma harzianum*, thereby further increasing the contents of glycyrrhizin and liquiritin (Xie et al., 2023a; Zhang et al., 2025). *Rhizophagus intraradices* in *Salvia miltiorrhiza* can significantly promote the accumulation of active components such as salvianolic acid B and tanshinone IIA (Zheng et al., 2023). In *Cannabis sativa*, inoculation with *Rhizophagus irregularis* can significantly improve the synthesis efficiency of phytocannabinoids such as cannabidivarin (CBDV) and cannabigerolic acid (CBGA) by optimizing the allocation of nitrogen and phosphorus nutrients (Ahmed et al., 2023).

Berberine bridge enzyme (BBE) in the alkaloid biosynthesis pathway, as a specific oxidase, has an essentially “oxidative coupling” catalytic mechanism. It requires oxygen (O_2_) as an electron acceptor to catalyze the cyclization reaction of (S)-reticuline, and this reaction is extremely sensitive to oxygen concentration (Dittrich and Kutchan, 1991). In contrast, *Variovorax* exhibits an extremely high respiratory rate in liquid medium. It is speculated that in the rhizomes of *C. chinensis*, *Variovorax* can regulate the redox state of the microenvironment by consuming a large amount of oxygen, leading to a decrease in rhizospheric oxygen concentration. This further inhibits BBE activity, impedes the conversion of (S)-reticuline (Kim et al., 2025), and ultimately reduces alkaloid content.

In addition, correlation analysis results showed that the content of columbamine exhibited a strong negative correlation with the relative abundances of *Streptomyces*, *Flavobacterium*, and *Rhizobium*; meanwhile, the content of palmatine showed a significant positive correlation with the relative abundance of *Paraburkholderia*. Combined with the analysis results of the *C. chinensis* alkaloid biosynthesis pathway, we speculate that *Streptomyces*, *Flavobacterium*, and *Rhizobium* may inhibit the conversion of (S)-tetrahydrocolumbamine to columbamine through direct or indirect pathways. For palmatine, its potential mechanism may be related to *Paraburkholderia* inducing the upregulation of the expression or enhancing the catalytic efficiency of caffeic acid O-methyltransferase (CoOMT), a key enzyme, thereby exerting positive regulation on the biosynthesis of palmatine. However, the above-mentioned mechanisms require further experimental verification.

#### 4.3.2 Associations between other differentially expressed metabolites and endophytes, and their ecological functions

Except for alkaloids, certain association mechanisms also exist between other differentially expressed metabolites (DEMs) and endophytes. Organic acids can not only regulate rhizospheric pH to create suitable colonization conditions for *Paraburkholderia* but also serve as easily utilizable carbon sources to support the growth and metabolism of this bacterium; phenylpropanoids can inhibit the growth of *Streptomyces* and reduce its infection risk (Bhattacharya et al., 2010). Therefore, the observed negative correlations between phenylpropanoids/polyketides and *Streptomyces* in this study may be a reflection of host metabolites inhibiting potentially harmful bacteria. Furthermore, polyketide synthase (PKS) genes provided by endophytes can assist in polyketide synthesis, while the host supplies precursors for phenylpropanoid synthesis, with the two synergistically enhancing the stress resistance of *C. chinensis*.

The positive correlation between lipid metabolites and the genus *Niastella*—which itself is significantly correlated with soil organic matter (SOM) and urease (URE) activity—indicates that along the altitude gradient, the low temperature and impoverished nutrient conditions at high altitudes will induce *C. chinensis* to accumulate more lipid metabolites. This further promotes the colonization and metabolic activity of *Niastella*, ultimately enhancing the adaptability of *C. chinensis* to high-altitude environments by optimizing nitrogen use efficiency.

### 4.4 Mediating roles of soil factors in the altitude-endophyte-alkaloid regulatory network

As a key geo-ecological factor, altitude affects environmental factors such as temperature, humidity, and light intensity (Holdsworth et al., 2025), thereby indirectly affecting soil physicochemical properties. The synergistic effect of these environmental factors and soil factors is the core driving force regulating plant secondary metabolism (Perrella et al., 2020). This study found that the rhizospheric soil physicochemical properties at different altitudes did not show a linear variation pattern with increasing altitude, but there were significant differences among different altitudes. This variation may be the outcome of the synergistic effect between soil environmental filtering and microbial communities, which indirectly regulates the growth of *C. chinensis* rhizomes and metabolite accumulation by affecting the composition of endophytic communities.

## 5 Conclusion

In summary, this study confirms that altitude regulates the yield, accumulation of active ingredients, and composition of endophytic microbial communities in *C. chinensis*. Within the studied altitudinal range, higher altitudes favor the enrichment of active ingredients as well as beneficial endophytic microbial communities. Although yield shows a slight decreasing trend with increasing altitude, the quality-related traits are significantly improved. Correlation analysis further indicates that altitudinally induced changes in the endophytic microbial communities are significantly associated with the accumulation of active ingredients in *C. chinensis*, thereby mediating the trade-off between yield and quality across different altitudinal gradients. These findings clarify the regulatory role of altitude in the quality formation and microecological adaptation of *C. chinensis*, providing a theoretical basis for its scientific cultivation and quality improvement.

## Data Availability

The datasets presented in this study can be found in online repositories. The names of the repository/repositories and accession number(s) can be found below: https://www.ncbi.nlm.nih.gov/, PRJNA1370352.
