# Supplementary material for: Altitudinal changes induce responses in Coptis chinensis Franch. rhizomes: endophytic communities, metabolite types, and alkaloid contents
Source: Front Plant Sci. 2026 Feb 23;17:1777206. doi: 10.3389/fpls.2026.1777206 (PMC12968305; doi:10.3389/fpls.2026.1777206)
Supplement: Supplementary file 1 [file DataSheet1.docx]

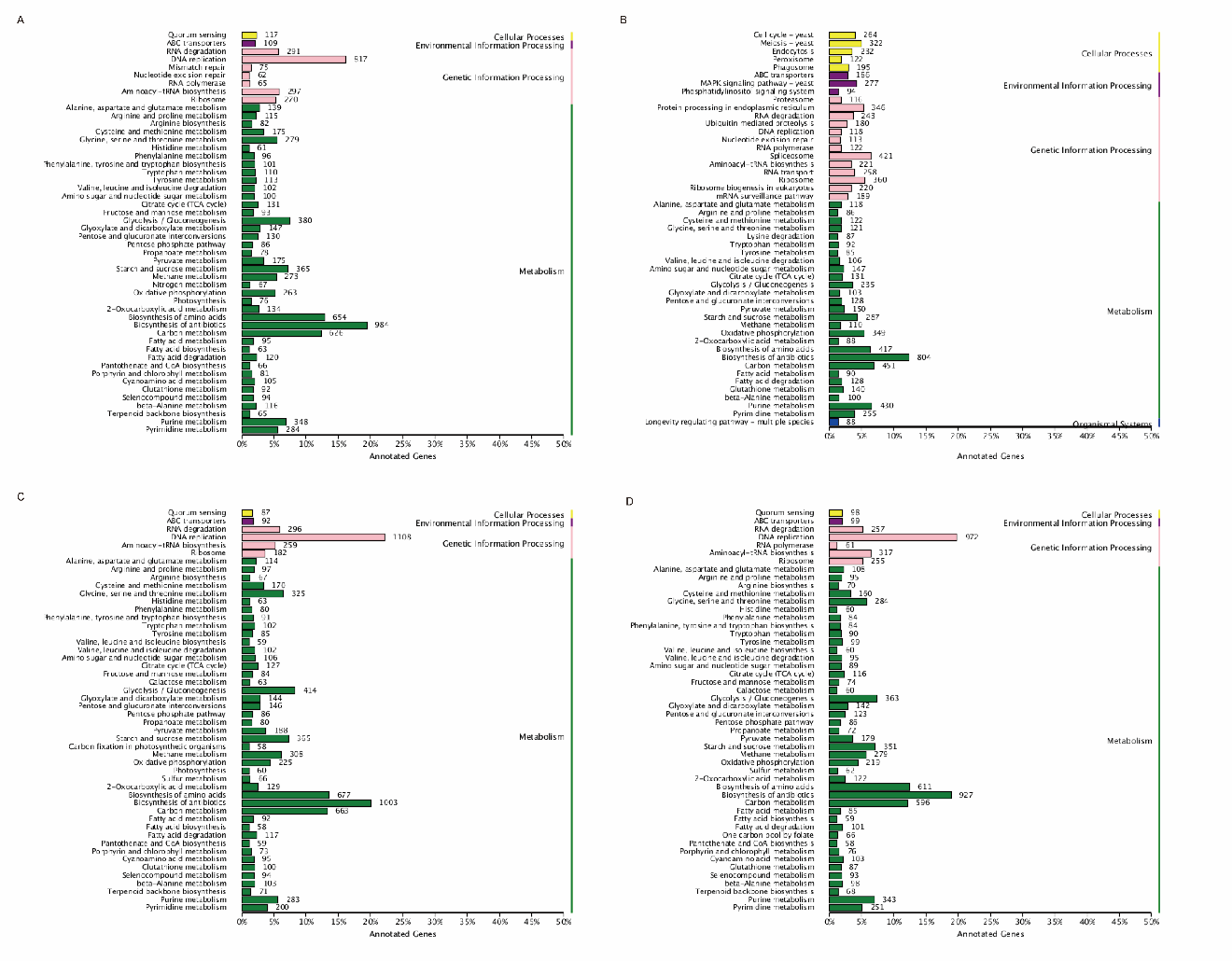


Fig. S1 Metagenomic KEGG functional annotation classification statistics of endophytic bacteria in *C. chinensis* rhizomes

The horizontal bar chart shows the KEGG pathway annotation results of endophytic bacterial metagenomes from *C. chinensis* samples of different altitude groups (A: HSA group; B: HSB group; C: HSC group; D: HSD group). The abscissa represents the number of annotated genes in each pathway, and the colored partitions on the right correspond to the four major categories of KEGG pathways (Cellular Processes, Environmental Information Processing, Genetic Information Processing, Metabolism). Bar segments of different colors represent specific pathways under each category; the same applies below.


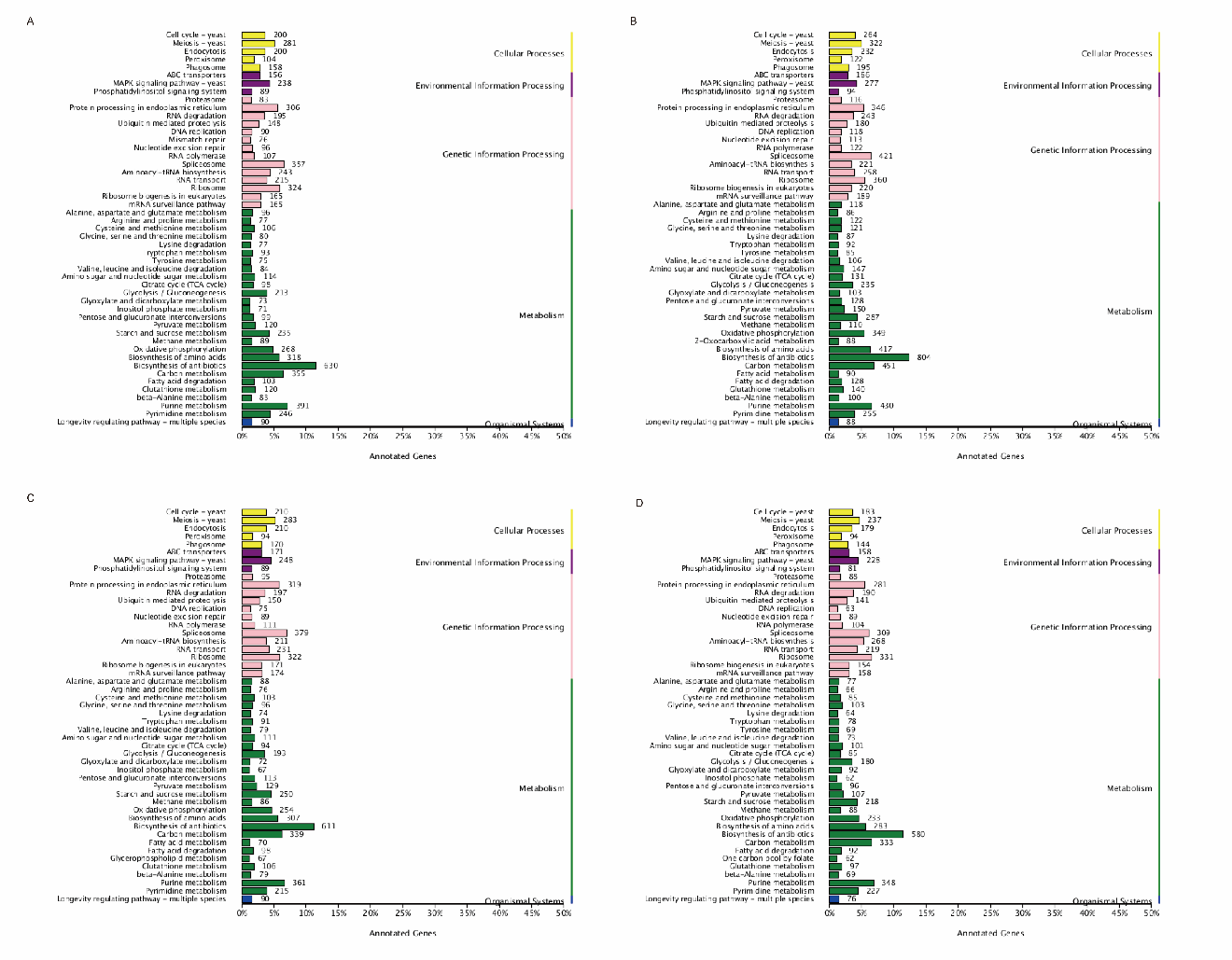


Fig. S2 Metagenomic KEGG functional annotation classification statistics of endophytic fungi in *Coptis chinensis* rhizomes

A: HSA group; B: HSB group; C: HSC group; D: HSD group
